# Differences in Personality Between High-Ability and Average-Ability University Students

**DOI:** 10.3390/jintelligence13010007

**Published:** 2025-01-07

**Authors:** Juan Francisco Flores-Bravo, Elena Rodríguez-Naveiras, María de los Dolores Valadez Sierra, Dylan Costantini, África Borges

**Affiliations:** 1 University Center of Health Sciences, University of Guadalajara, Guadalajara 44340, Mexico; francisco.flores@academico.udg.mx; 2 Department of Clinical Psychology, Psychobiology and Methodology, University of La Laguna, 38200 San Cristóbal de La Laguna, Spain; naveiras@ull.edu.es (E.R.-N.); aborges@ull.edu.es (Á.B.); 3 Working and Research Group on Giftedness, University of La Laguna, Santa Cruz de Tenerife, 38200 San Cristóbal de La Laguna, Spain; dylan.dor.2019@gmail.com

**Keywords:** high abilities, average ability, university students, personality

## Abstract

Despite the growing body of research examining the personality traits of individuals with high abilities, little clarity exists about how they differ from the general population, especially within the university context. This study aimed to identify distinct personality traits by examining 268 high-ability university students alongside a matched average-ability group through a retrospective ex post facto design. Results revealed significant differences only in the trait of responsibility (*p* = 0.037), with lower scores observed among high-ability students. This outcome may be related to specific academic environmental factors, such as insufficient challenges. It can be concluded that, although stereotypes often associate high-ability students with certain personality traits, such as openness to experience, the present results do not reflect such differences. Therefore, it is important to conduct well-designed studies to determine the characteristics of high-ability individuals and how they differ from average-ability students.

## 1. Introduction

Starting university requires a profound process of emotional, social, and academic integration (Padilla González et al. 2017), as it represents a key stage in students’ development (Almukhambetova and Hernández-Torrano 2020). For Latin American university students, social and institutional conditions can contribute to stress, depression, anxiety, and uncertainty (Parra 2021). Factors such as career choice can also influence these conditions (Caro et al. 2019).

This reality does not exclude university students with high abilities, a group frequently invisibilized in part by the false belief that they do not have special educational needs and that their success is assured (Almukhambetova and Hernández-Torrano 2020). From the perspective of Gagné (2015, 2021) and his Comprehensive Model of Talent Development (CMTD), high abilities are understood as natural and innate abilities that, when worked systematically in a facilitating environment, can lead to the development of talent.

In this model, the author establishes a key distinction between giftedness and talent: Giftedness (aptitudes) refers to the spontaneous manifestation of superior natural abilities that have not been systematically trained but emerge from interaction with the environment. Talent, on the other hand, refers to those capabilities (competencies) that have been systematically developed through a structured process. In both cases, both gift and talent position the individual within the top 10% compared to his or her peers (Gagné 2021). Under this perspective, talent development does not occur in isolation but is the result of the dynamic interaction between personal factors (such as motivation and perseverance) and contextual factors (such as family and educational environment), which emphasizes the importance of creating favorable conditions that allow high abilities to transform into effective talent (Gagné 2021).

Thanks to its influence and wide international recognition during the last decade, this model is adopted as a fundamental reference in the present study for the conceptualization and care of the population with high abilities.

Likewise, an important reference for the assessment of students with high abilities is the Tripartite Model of High Ability proposed by Pfeiffer (2013, 2015). This model integrates developmental, psychometric, and transformational approaches, offering three perspectives for understanding high ability: (1) as high intelligence, (2) as exceptional performance, and (3) as high potential to excel.

This study adopts the first perspective of the tripartite model, which conceives of high ability as high intelligence. From this approach, the identification of students with high intellectual abilities can be performed through the application of intelligence quotient (IQ) tests, cognitive ability assessments, or equivalent instruments that are rigorous and scientifically valid. This perspective can be approached from a general view (g) of intelligence or from a multidimensional approach, depending on the assessment criteria and tools used.

Understanding the characteristics of high-ability individuals is therefore essential. Although interest in this area is growing, research on adults remains limited, particularly in the study of socio-emotional and personality traits. This topic is particularly controversial within the university context, given the variety of perspectives on the subject (Casino-García et al. 2021; Papadopoulos 2020).

Socio-emotional aspects remain a topic of debate. Some studies suggest that gifted students exhibit appropriate socio-emotional adjustment, often using cognitive skills to solve problems that allow them to socially adapt at a level comparable to their peers (Borges et al. 2011; Casino-García et al. 2021; Franklin et al. 2016; Rodríguez-Naveiras et al. 2018; Segaran and Hasim 2021; Sternberg 2020; Valadez Sierra et al. 2016). However, other studies point to socio-emotional maladjustment, identifying risks such as fear of failure, anxiety, helplessness, and low self-esteem (Casino-García et al. 2021; Day et al. 2015; Fraser et al. 2014).

Personality traits—consistent patterns of thoughts, emotions, and behaviors shaped by cultural norms—vary with socio-cultural context (Ogurlu and Özbey 2021). Recent theories on giftedness consider socio-emotional constructs in studying personality traits. For example, Renzulli (2005) posits that motivation is a core component of giftedness, along with above-average intellectual ability and creativity. In this model, motivation is associated with traits such as persistence, resilience, and self-confidence (Renzulli 2012).

Among the various theories of personality, the Five Factor Model (McCrae and Costa 1987; McCrae and Costa 1999) is widely accepted to explain individual differences in adapting to and interacting with our environment. This model includes five dimensions: openness to experience, extraversion, agreeableness, conscientiousness, and neuroticism.

Openness to experience involves curiosity, originality, creativity, and a desire for new experiences; extraversion encompasses energy, sociability, assertiveness, and enjoyment of social interaction; agreeableness includes empathy and trust; conscientiousness involves organization, attention to detail, and reliability; and neuroticism encompasses negative emotions like anxiety, nervousness, anger, depression, and vulnerability (Ogurlu and Özbey 2021).

A topic of great interest and debate is the association between intelligence and personality (Fries et al. 2022), as well as the changes that the latter undergoes throughout life. This debate arises, to a large extent, from the multiple reasons that may explain the contradictory findings on the personality of adults with high abilities. Among these reasons are methodological differences, such as the conduct of correlational studies comparing the general population with those that include groups of high abilities, as well as different inclusion criteria for sample selection. These criteria may differ with respect to IQ threshold, academic achievement, or membership in associations specializing in high abilities and talents. Added to this is the absence of a unified theoretical framework, which hinders the consistent interpretation of the results (Matta et al. 2019).

Previous research has mostly addressed this relationship through correlational designs, which have focused on modeling linear associations between both variables (Chamorro-Premuzic and Furnham 2008; Kretzschmar et al. 2018). However, the question arises: what about the most able? Given that this group could show behavioral and association patterns different from those observed in individuals with average intelligence (Matta et al. 2019).

Specialized literature highlights personality traits that both favor and disfavor high-ability students, with most studies focusing on pre-university levels. Some studies recognize that high-ability individuals differ from the general population in certain personality aspects, such as greater openness to experience, which is more strongly associated with intelligence than other personality dimensions, as shown in previous studies (De Gucht et al. 2023; DeYoung 2011; Stanek 2014).

These findings are consistent with the study by Ogurlu and Özbey (2021), who conducted a meta-analysis examining personality differences between individuals with high and low abilities and found that high-ability individuals tended to score higher on openness to experience (g = 0.473).

In addition, Frumau-Van Pinxten et al. (2021) identified openness to experience as a key characteristic of high-ability adolescents, associated with high developmental potential. However, participants also showed comparable levels of emotional stability and agreeableness to their high-ability peers, suggesting that while ability may enhance certain positive traits, it does not guarantee greater emotional well-being.

Fries et al. (2022) also studied adults in the Mensa International Society using the HEXACO model, finding significant scores in openness to experience and other traits like honesty-humility and conscientiousness, with lower emotionality, indicating a structured, achievement-oriented personality, though low emotionality may indicate an area for improvement.

In another recent study, De Gucht et al. (2023) found significantly higher scores in openness to experience and lower neuroticism in high-ability adults compared to the general population.

Mammadov (2022) identified four personality profiles in adolescents: resilient, average, overcontrolled, and introverted. The resilient group, the largest, showed a favorable pattern of personality traits, with high life satisfaction, strong social support, and academic success, highlighting the positive impact of high ability on personal and academic development. However, other profiles face specific challenges: overcontrolled individuals may struggle emotionally, while introverts may experience limitations.

In summary, openness to experience stands out as one of the most evidenced personality dimensions in the specialized literature, partly because this personality trait has been mostly studied due to its prominent role in two fundamental theories: Cattell’s (1987) investment theory and Ziegler et al. (2012) OFCI model, which provide a theoretical framework for understanding how personality and intelligence interact.

Cattell’s investment theory states that fluid intelligence (Gf), understood as the innate ability to reason, solve abstract problems and adapt to new situations, forms the basis of crystallized intelligence (Gc), which is presented as the set of knowledge and skills acquired throughout life through educational contexts, acquired experiences and the culture in which a person is immersed. Crystallized intelligence develops through the investment of fluid intelligence in specific contexts (Cattell 1987).

Under this theoretical model, openness to experience plays a facilitating role in allowing people to channel their fluid intelligence into activities that promote the development of crystallized intelligence. This process occurs because people with high levels of openness tend to seek intellectual, cultural and educational experiences, which enriches their knowledge base and strengthens skills related to crystallized intelligence.

Based on the investment theory of Cattell (1987) and Ackerman (1996), Ziegler et al. (2012) developed the Openness-Fluid-Crystallized Intelligence (OFCI) model, which extends the relationship between fluid intelligence and crystallized intelligence, further highlighting the central role of openness as a key factor in this interaction. This model includes the environmental enrichment hypothesis, which states that people with higher levels of openness tend to seek and expose themselves to new experiences and learning, which not only strengthen their fluid intelligence by exercising their intellectual skills but also indirectly contribute to the development of crystallized intelligence by transforming novel information into accumulated knowledge. Longitudinal studies have supported this relationship, evidencing the influence of openness on the development of fluid intelligence in different contexts (Trapp et al. 2019; Ziegler et al. 2012; Ziegler et al. 2015).

Personality differences between the adult population with high abilities and the general population have also been the subject of study, considering the gender variable. However, specific research in this regard is limited.

As previously noted, in general, it has been observed that people with high abilities tend to score higher on the “Openness to Experience” trait, regardless of gender (Ogurlu and Özbey 2021), without finding significant differences in other personality traits, such as Agreeableness, Extraversion, Scrupulousness or Neuroticism, when comparing people with high abilities with the general population.

On the other hand, regarding personality differences according to gender in university populations of average intelligence, some studies indicate that women tend to score higher in the dimensions of Neuroticism and Agreeableness, while men tend to score higher in Extraversion and Scrupulosity (Rouco et al. 2014). Meanwhile, studies conducted at pre-university levels also conclude that students with high abilities present a homogeneous personality profile as do their peers of average intelligence (Trillo Luque 2012; Sánchez 2006).

As can be seen, there are no conclusive studies that confirm differences in personality and gender with respect to university students with high abilities in relation to average-ability students.

In view of the scarcity of research on personality characteristics in university students with high abilities, the present study aims to verify the veracity of the personality traits that distinguish these students from those of average ability, based on the hypothesis that people with high abilities are more open to experience.

## 2. Materials and Methods

### 2.1. Methodology and Design

This study used a retrospective ex post facto design, comparing high-ability students with those of average ability.

### 2.2. Participants

Sampling was carried out in two stages. In the first phase, a convenience sample was obtained by inviting all first-semester students at the University Center of Health Sciences (CUCS) at the University of Guadalajara who enrolled during the 2023B and 2024A school terms. Of the 2900 students, 2746 agreed to participate, who made up the general sample. The call included the undergraduate programs in Forensic Sciences, Dental Surgeon, Physical Culture and Sports, Nursing, Physical Therapy, Surgeon and Midwife, Nutrition, Podiatry and Psychology, as well as the undergraduate programs in Dental Prosthesis, Radiology and Imaging, and Emergency, Occupational Safety and Rescue.

In the second phase, students were selected from the general sample who, according to Cattell and Pfeiffer’s criteria, had an IQ equal to or higher than 120, for a total of 268 participants, who made up the sample of high abilities. Subsequently, a group of students with an IQ close to 100, representative of average intelligence, was chosen, equaling the number of members of the high-ability sample and ensuring correspondence in gender, career and age. Thus, the average-ability sample was formed (see Table 1).

### 2.3. Instruments

The following instruments were used in the study, with the permission of the respective authors. A detailed description of each tool, including its main characteristics and psychometric properties, is given below.

(a) RRHH Matrices, Test for the Identification of Talent and Learning Potential (Sánchez-Sánchez et al. 2020): It is a newly created test that aims to assess reasoning ability and problem-solving skills by means of the visual representation of matrices, an aspect widely investigated and employed for decades both in the applied and research fields. This type of task facilitates an accurate estimation of the ability to solve complex and novel problems, a skill closely linked to the Gf factor, or fluid intelligence, which in turn is one of the best indicators of the general ability or g factor within the Cattell (1987) model. In each exercise, a matrix composed of nine elements, organized in three rows and three columns, must be analyzed to discover the logical pattern underlying the different figures. This type of activity is associated with one of the best predictors of general intelligence capacity. Its application is online, which makes it possible to obtain the results immediately, together with a short report in which the results are analyzed individually; it can be both individual and collective in adults from 18 years of age in an estimated time of 30 min. It presents high reliability (α = 0.82) and convergent validity (latent correlation: r = 0.95), as demonstrated by confirmatory factor analysis with other similar tests such as the *Reynolds Intellectual Assessment Scales* (RIAS; Reynolds and Kamphaus 2009) and the *TEA’s Aptitude Battery* (BAT-7; Arribas et al. 2013). Overlapping samples were used, controlling for several variables such as place of residence, educational level, age, sex, among others, which favors the generalization of its results (Sánchez-Sánchez et al. 2020). These values reflect high accuracy in the RRHH Matrices for predicting performance on abstract reasoning and problem-solving tasks.
  The RRHH Matrices test has become a widely used tool due to its innovative features. In addition to integrating the traditional properties of a group-application intelligence test, it stands out for incorporating Item Response Theory (IRT) and for using a computerized adaptive test format as one of its main features, which makes it easy for each participant to receive his or her own test.
(b) Occupational Personality Assessment (O-PER-A, Hell and Päßler, forthcoming): It assesses fundamental personality traits relevant to the work environment. This instrument is based on the principles of widely recognized models, such as the Big Five, and also incorporates key findings related to the Dark Triad (narcissism, subclinical psychopathy and Machiavellianism) and the H factor of the HEXACO model, which measures dimensions such as honesty and modesty. It consists of 129 items on a Likert-type scale ranging from 1 = not at all to 6 = very much. The remaining response options (2, 3, 4 and 5) are not labeled for the degree to which they describe the person evaluated. Its design allows for a comprehensive analysis that combines positive and challenging traits of human behavior through six global dimension scales, which, in turn, contain two specific scales per dimension: Stability (Resilience and Balance), Extraversion (Assertiveness and Sociability), Openness (Openness to Novelty and Aesthetics), Kindness (Helpfulness and Empathy and Cooperation), Responsibility (Achievement Motivation and Planning) and Integrity (Honesty and Humility). The internal consistency reliability ranges between 0.79 and 0.89, while test-retest reliability is between 0.64 and 0.89.

### 2.4. Procedure

The study adhered to ethical guidelines for data handling and protection, following the CIOMS (2016) International Ethical Guidelines for Health-related Research, the Mexican Federal Law on Data Protection (Diario Oficial de la Federación 2010), and the American Psychological Association (2024) Ethical Principles for Psychologists. Data collection took place in August 2023 (2023B term) and January 2024 (2024A term), using online tests administered through the TEA publishing portal, in the presence of qualified raters.

The research team at the CUCS Institute of Psychology and Special Education issued the call for the study and promoted it through undergraduate program coordinators and official CUCS websites. Likewise, the benefits of participating in the study were made known, such as having a profile of their socio-affective characteristics and being part of a mentoring program for the attention of students with high abilities, in case they have such a profile, in addition to receiving attention from other agencies when areas of opportunity are detected.

Participation was voluntary and informed consent was obtained beforehand to ensure confidentiality and to clarify that participation would not affect students’ grades or standing.

### 2.5. Data Analysis

Multivariate analysis of variance (MANOVA) was conducted using SPSS version 25 to determine if there were personality differences between high-ability students and the average-ability sample.

## 3. Results

Reliability was first calculated for this sample. Table 2 shows the reliability scores for each dimension of the Test Occupational Personality Assessment (O-PER-A).

The reliability of the Matrices-TAI test was measured with an ordinal alpha of 0.87 (CI 0.81–0.93) and an ordinal omega of 0.88 (CI 0.82–0.94).

A MANOVA was performed on the six personality dimensions included in the O-PER-A test to examine differences between the groups. Descriptive statistics are presented in Table 3.

The homogeneity of the covariance matrices was tested, supporting the null hypothesis of no differences between the observed covariance matrices of the dependent variables across groups (Box’s M = 73.202, F(63, 584,047.492) = 1.138, Sig. = 0.212). Levene’s test for homoscedasticity was also applied (see Appendix A).

Wilks’ Lambda was used to ensure robust results in the Responsibility dimension, ensuring greater precision between the groups analyzed (see Table 4).

Due to significant differences between groups, univariate between-subjects tests for high ability are shown in Table 5, with the only significant effect found on the “Responsibility” dimension. However, the effect size does not allow for a conclusive explanation of the differences.

Table 6 shows the between-subjects gender effects.

## 4. Discussion

The purpose of this study was to examine the personality traits that distinguish high-ability university students from an average-ability sample, given the limited literature exploring personality differences in adult populations. Understanding these distinguishing traits is essential to clarify what truly characterizes individuals with high intellectual ability.

The results indicate that high-ability university students scored significantly lower than the average-ability sample on the Responsibility dimension—this being the only variable that showed significant differences between the two groups. Lower responsibility may reflect these students’ dissatisfaction with previous educational experiences, as suggested by Rocha et al. (2024). Although the differences in Responsibility are significant, the very small effect size means that these findings should be interpreted with caution and need to be replicated in other samples.

These results are consistent with other studies that have found significant differences in only one personality dimension. For example, Ogurlu and Özbey (2021) found greater Openness to Experience in high-ability students compared to their peers. This highlights that high-ability students do not exhibit negative traits across all personality dimensions, challenging social and educational stereotypes that associate high ability with inferior emotional and social skills (Baudson and Preckel 2016; Preckel et al. 2015).

Further research into personality dimension differences is essential, as the results of the current study are inconsistent with previous findings that identified Openness to Experience as a distinguishing factor in this population, while differences were found in Responsibility. However, the effect size was not significant. It is crucial to establish the extent to which high-ability individuals differ from the average-ability sample, which should be supported by studies characterized by rigorous methodology, including a control group.

When analyzing personality differences by gender in the general sample, the results show that women score higher on Responsibility, Extraversion, Agreeableness, Integrity, and Openness, and lower on Stability. These differences may be explained by differences in psychological development, gender roles, and cultural factors that influence how personality is expressed in men and women (Pirlott and Schmitt 2014; Schmitt et al. 2017).

The dimensions on which women scored higher can be contextualized as follows: Responsibility may be influenced by societal expectations that assign them a task-oriented role in both family and professional settings (Schmitt et al. 2017), which pushes women to be meticulous and reliable. Extraversion is fostered in many cultures, encouraging women to build social relationships and develop interpersonal skills (Contreras and Flores 2022). Agreeableness is often linked to cultural expectations for women to prioritize the emotional needs of others and demonstrate empathy (Schmitt et al. 2017). Integrity may relate to the expectations of consistency between their values and behaviors (Ogurlu and Özbey 2021), and Openness reflects women’s interest in exploring new ideas and engaging in creative activities.

However, women scored lower on Stability, possibly because they often face greater emotional demands and multiple roles, such as family care, work responsibilities, and academics, which may increase exposure to stress and anxiety (Schmitt et al. 2017).

Furthermore, the results show no interaction between ability level and gender. This suggests that high-ability students behave similarly regardless of gender, as no significant interaction was found.

These findings challenge common stereotypes that associate high-ability students with gender-specific personality traits.

One of the main strengths of this study is the sample size, as research with high-ability students often involves smaller samples, particularly in university contexts where there are no standardized frameworks to facilitate identification. However, a limitation of this research is that all participants are from health sciences programs, which limits the generalizability of the findings to other fields. Including students from diverse academic fields would yield more robust results.

Another limitation of this study lies in the use of the O-PER-A questionnaire, an instrument specifically designed to assess personality dimensions in the occupational context. Although the O-PER-A is based on solid theoretical models such as the Big Five and the HEXACO model, and collects dimensions relevant to understanding human personality in general, its main focus is oriented towards characteristics associated with occupational performance. Therefore, when applied to university students, who are in a stage prior to formal insertion in the workplace, it is possible that some of the dimensions assessed do not fully reflect the personality profile of this group.

In addition, it is important to consider that the interpretation of the results could be limited by the lack of specific adaptations to the university context, which could restrict the generalization of the results outside the occupational setting.

The similarity between the groups of participants may also explain the results; although the control group differs in intelligence, it may share similarities with the gifted group in that it is close to the threshold of academic talent. This is because the educational programs offered at the Centro Universitario de Ciencias de la Salud are highly competitive and require the highest admission scores at the University of Guadalajara.

These findings would therefore be strengthened by replicating this study with a larger, more diverse sample in a different geographical context. However, this is a considerable challenge and would require a considerable investment in material and human resources.

Despite this limitation, the findings provide valuable insights into the personality characteristics of high-ability students in a university setting. Exploring these dimensions helps to dispel stereotypes and promotes a more holistic approach to addressing the educational needs of this group.

In this context, the present study provides valuable information that reinforces the truth of the personality traits of high-ability students and contributes to a theoretically grounded framework in university contexts, which is essential for identifying high-ability individuals even at advanced stages of life. This identification is crucial for designing interventions that meet their needs (Nalevaiko Rocha et al. 2021).

In Mexico, although the Ministry of Public Education (SEP 2006, 2022) has developed an Educational Support Proposal for Students with High Abilities, focusing on enrichment models, this only applies to primary education, leaving a gap in continuity at the high school and university levels. Given this scenario, there is a need to extend support for students with high abilities in higher education, equipping teachers with an inclusive mindset towards high abilities (Reynen-Woodward et al. 2023), and providing guidance to families (Storrer de Oliveira and Joaquim Minetto 2021), as high abilities do not automatically guarantee personal or professional success in adulthood (Ozcan 2017). Therefore, it is crucial to guide students with high abilities with realistic expectations, regardless of their age.

Due to the inconsistencies in the literature on personality differences between high-ability university students and their peers, as well as certain misconceptions about the personalities of high ability individuals, further research is needed to clarify the knowledge gap in this area. As Brown and Peterson (2022) point out, a better understanding of this population will contribute to the greater well-being of high-ability adults in general.

## Figures and Tables

**Table 1:** Characteristics of Participants.

**High-Ability Sample**			
	**Participants**	**Mean IQ (Range)**	**Mean Age (S.D.) (in Years)**
Men	115	126.35 (120–144)	19.67 (2.04)
Women	153	125.50 (120–139)	19.65 (2.03)
Total	268	125.92 (120–144)	19.66 (2.04)
**Average-Ability Sample**			
	**Participants**	**Mean IQ (Range)**	**Mean Age (S.D.) (in Years)**
Men	116	96.84 (82–109)	19.66 (2.00)
Women	152	97.28 (82–104)	19.64 (2.00)
Total	268	97.06 (82–109)	19.65 (2.00)

**Table 2:** Reliability of Dimensions of Test Occupational Personality Assessment (O-PER-A) Calculated for the Sample.

Dimensions O-PER-A	α
Responsibility	0.89
Stability	0.88
Extraversion	0.76
Agreeableness	0.86
Integrity	0.89
Openness	0.86

**Table 3:** Descriptive Statistics by Sample Groups.

Dimensions	High-Ability Sample				Average-Ability Sample			
	Men		Women		Men		Women	
	Mean	SD	Mean	SD	Mean	SD	Mean	SD
Responsibility	89.31	13.71	92.44	13.67	91.97	13.90	94.37	11.31
Stability	66.34	13.34	57.68	14.86	64.57	13.51	55.83	14.52
Extraversion	68.97	11.93	69.86	11.06	71.59	11.39	70.12	10.73
Agreeableness	85.46	11.71	88.27	11.67	82.56	11.64	88.10	11.96
Integrity	103.42	18.47	105.42	18.17	99.35	19.52	105.81	18.13
Openness	75.22	11.80	78.27	9.66	74.34	10.48	76.52	10.89

**Table 4:** Effects Obtained in MANOVA on Independent Variables and Their Interaction.

Effects	Lambda of Wilks	F (6, 527)	Sig.	Partial η^2^
High Abilities	0.963	3.351	0.003	0.037
Gender	0.846	16.028	0.000	0.154
High Abilities-Gender	0.990	0.895	0.498	0.010

**Table 5:** Tests of Between-Subjects Effects—High Abilities.

Dimensions	Mean Square	F (1, 532)	Sig.	Partial η^2^
Responsibility	690.217	4.015	0.046	0.007
Stability	430.851	2.148	0.143	0.004
Extraversion	271.523	2.151	0.143	0.004
Agreeableness	309.674	2.240	0.135	0.004
Integrity	444.912	1.296	0.255	0.002
Openness	225.709	1.980	0.160	0.004

**Table 6:** Tests of Between-Subjects Effects—Gender.

Dimensions	Mean Square	F(1, 532)	Sig.	Partial η^2^
Responsibility	1008.878	5.869	0.016	0.011
Stability	9948.313	49.589	0.000	0.085
Extraversion	11.265	0.089	0.765	0.000
Agreeableness	2288.649	16.556	0.000	0.030
Integrity	2353.696	6.857	0.009	0.013
Openness	897.294	7.873	0.005	0.015

## Data Availability

This study was conducted in strict compliance with the General Law for the Protection of Personal Data in Possession of Obligated Subjects of Mexico. In this context, it is reported that the data received electronically are stored on the hard disk of an institutional PC of the University Center of Health Sciences of the University of Guadalajara. Due to the sen-sitive nature of the information, its public disclosure is not possible.

## Appendix A

**Table A1:** O-PER-A Homoscedasticity.

Dimensions	Levene’s Statistic (3, 532)	Sig.
Responsibility	2.855	0.037
Stability	1.092	0.352
Extraversion	1.074	0.360
Agreeableness	0.113	0.953
Integrity	0.633	0.594
Openness	2.359	0.071
